# 2-Methyl­benzimidazolium thio­cyanate

**DOI:** 10.1107/S1600536810042145

**Published:** 2010-10-23

**Authors:** Shayma A. Shaker, Hamid Khaledi, Hapipah Mohd Ali

**Affiliations:** a Department of Chemistry, University of Malaya, 50603 Kuala Lumpur, Malaysia

## Abstract

In the crystal structure of the title compound, C_8_H_9_N_2_^+^·SCN^−^, the nearly planar 2-methyl­benzimidazolium cation [r.m.s. deviation = 0.0123 (4) Å] is perpendicular to a mirror plane and the methyl H atoms are disordered about the mirror plane with equal occupancies. The thio­cyanate anion also lies on a mirror plane. N—H⋯N hydrogen bonds link the components into an infinite chain along the *b* axis.

## Related literature

For related structures, see: Bhattacharya *et al.* (2004 ▶); Ding *et al.* (2004 ▶); Shaker *et al.* (2010 ▶); Huang *et al.* (2006 ▶). For the application of benzimidazole derivatives in crystal engineering, see: Cai *et al.* (2002 ▶). For the biological properties of benzimidazole derivatives, see: Refaat (2010 ▶); Ansari & Lal (2009 ▶).
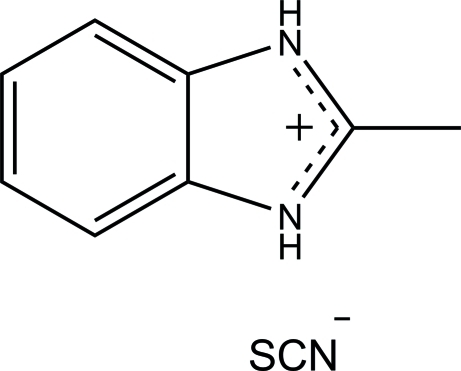


## Experimental

### 

#### Crystal data

C_8_H_9_N_2_^+^·SCN^−^
*M* *_r_* = 191.25
Orthorhombic, 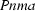

*a* = 9.879 (2) Å
*b* = 7.2157 (15) Å
*c* = 12.890 (3) Å
*V* = 918.9 (3) Å^3^
*Z* = 4
Mo *K*α radiation
μ = 0.31 mm^−1^
*T* = 100 K
0.40 × 0.29 × 0.15 mm

#### Data collection

Bruker APEXII CCD diffractometer
Absorption correction: multi-scan (*SADABS*; Sheldrick, 1996 ▶) *T*_min_ = 0.888, *T*_max_ = 0.956
10495 measured reflections
1133 independent reflections
1000 reflections with *I* > 2σ(*I*)
*R*_int_ = 0.039

#### Refinement

*R*[*F* ^2^ > 2σ(*F* ^2^)] = 0.034
*wR*(*F* ^2^) = 0.090
*S* = 1.01
1133 reflections
71 parameters
1 restraint
H atoms treated by a mixture of independent and constrained refinement
Δρ_max_ = 0.39 e Å^−3^
Δρ_min_ = −0.28 e Å^−3^

### 

Data collection: *APEX2* (Bruker, 2007 ▶); cell refinement: *SAINT* (Bruker, 2007 ▶); data reduction: *SAINT*; program(s) used to solve structure: *SHELXS97* (Sheldrick, 2008 ▶); program(s) used to refine structure: *SHELXL97* (Sheldrick, 2008 ▶); molecular graphics: *X-SEED* (Barbour, 2001 ▶); software used to prepare material for publication: *SHELXL97* and *publCIF* (Westrip, 2010 ▶).

## Supplementary Material

Crystal structure: contains datablocks I, global. DOI: 10.1107/S1600536810042145/is2616sup1.cif

Structure factors: contains datablocks I. DOI: 10.1107/S1600536810042145/is2616Isup2.hkl

Additional supplementary materials:  crystallographic information; 3D view; checkCIF report

## Figures and Tables

**Table 1:** Hydrogen-bond geometry (Å, °)

*D*—H⋯*A*	*D*—H	H⋯*A*	*D*⋯*A*	*D*—H⋯*A*
N2—H2⋯N1	0.88 (2)	2.00 (2)	2.8627 (16)	168 (2)

**Fig. 1.**
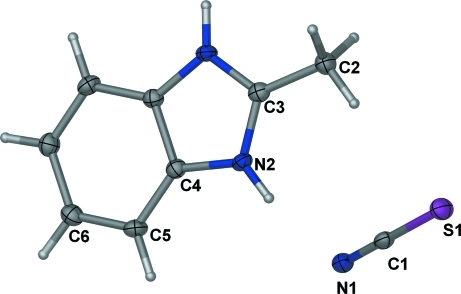
Thermal ellipsoid plot of the title compound at the 50% probability level. Hydrogen atoms are drawn as spheres of arbitrary radius. The unlabelled atoms are generated by the symmetry operation (x, –y + 3/2, z).

## supplementary crystallographic information

### Comment

Benzimidazoles are a class of compounds with a wide variety of biological properties (Refaat, 2010; Ansari & Lal, 2009) and applications in crystal-engineering (Cai *et al.*, 2002). During our studies on coordination behavior of 2-methylbenzimidazole, the title crystal was obtained unexpectedly as a by-product. The structures of several compounds similar to present structure have been reported (Bhattacharya *et al.*, 2004; Ding *et al.*, 2004; Shaker *et al.*, 2010; Huang *et al.*, 2006).

The asymmetric unit of the title compound, contains one-half molecule of each component. The nearly planar 2-methylbenzimidazolium moiety (r.m.s = 0.0123 Å) is perpendicular to, and the thiocyanate ion lies on a mirror plane. In the crystal structure, an N—H···N hydrogen bond links the molecules into an infinite chain along the *b* axis.

### Experimental

An ethanolic solution (12 ml) of 2-methylbenzimidazole (5 mmol, 0.78 g) was added to an aqueous solution (10 ml) of CuCl_2_. 2H_2_O (0.5 g, 2 mmol) followed by addition of an aqueous solution (10 ml) of KSCN (5 mmol).The resulting precipitates were filtered off. The colorless crystals of the title compound were obtained from the filtrate.

### Refinement

The C-bound hydrogen atoms were placed at calculated positions (C—H 0.95 or 0.98 Å) and were treated as riding on their parent atoms, with *U*_iso_(H) set to 1.2 or 1.5 *U*_eq_(C). The N-bound hydrogen atom was located in a difference Fourier map and refined with a distance restraint of N—H 0.88 (2) Å.

### Crystal data

**Table:**

C_8_H_9_N_2_^+^·SCN^−^	*F*(000) = 400
*M**_r_* = 191.25	*D*_x_ = 1.382 Mg m^−^^3^
Orthorhombic, *P**n**m**a*	Mo *K*α radiation, λ = 0.71073 Å
Hall symbol: -P 2ac 2n	Cell parameters from 4285 reflections
*a* = 9.879 (2) Å	θ = 2.6–30.3°
*b* = 7.2157 (15) Å	µ = 0.31 mm^−^^1^
*c* = 12.890 (3) Å	*T* = 100 K
*V* = 918.9 (3) Å^3^	Block, colorless
*Z* = 4	0.40 × 0.29 × 0.15 mm

### Data collection

**Table:**

Bruker APEXII CCD diffractometer	1133 independent reflections
Radiation source: fine-focus sealed tube	1000 reflections with *I* > 2σ(*I*)
graphite	*R*_int_ = 0.039
φ and ω scans	θ_max_ = 27.5°, θ_min_ = 2.6°
Absorption correction: multi-scan (*SADABS*; Sheldrick, 1996)	*h* = −12→12
*T*_min_ = 0.888, *T*_max_ = 0.956	*k* = −9→9
10495 measured reflections	*l* = −16→16

### Refinement

**Table:**

Refinement on *F*^2^	Primary atom site location: structure-invariant direct methods
Least-squares matrix: full	Secondary atom site location: difference Fourier map
*R*[*F*^2^ > 2σ(*F*^2^)] = 0.034	Hydrogen site location: inferred from neighbouring sites
*wR*(*F*^2^) = 0.090	H atoms treated by a mixture of independent and constrained refinement
*S* = 1.00	*w* = 1/[σ^2^(*F*_o_^2^) + (0.0395*P*)^2^ + 0.9286*P*] where *P* = (*F*_o_^2^ + 2*F*_c_^2^)/3
1133 reflections	(Δ/σ)_max_ < 0.001
71 parameters	Δρ_max_ = 0.39 e Å^−^^3^
1 restraint	Δρ_min_ = −0.28 e Å^−^^3^

### Special details

**Table:**

Geometry. All e.s.d.'s (except the e.s.d. in the dihedral angle between two l.s. planes) are estimated using the full covariance matrix. The cell e.s.d.'s are taken into account individually in the estimation of e.s.d.'s in distances, angles and torsion angles; correlations between e.s.d.'s in cell parameters are only used when they are defined by crystal symmetry. An approximate (isotropic) treatment of cell e.s.d.'s is used for estimating e.s.d.'s involving l.s. planes.
Refinement. Refinement of *F*^2^ against ALL reflections. The weighted *R*-factor *wR* and goodness of fit *S* are based on *F*^2^, conventional *R*-factors *R* are based on *F*, with *F* set to zero for negative *F*^2^. The threshold expression of *F*^2^ > σ(*F*^2^) is used only for calculating *R*-factors(gt) *etc*. and is not relevant to the choice of reflections for refinement. *R*-factors based on *F*^2^ are statistically about twice as large as those based on *F*, and *R*- factors based on ALL data will be even larger.

### Fractional atomic coordinates and isotropic or equivalent isotropic displacement parameters (Å^2^)

**Table:**

	*x*	*y*	*z*	*U*_iso_*/*U*_eq_	Occ. (<1)
S1	−0.11964 (6)	0.2500	0.31732 (5)	0.02094 (18)	
N1	0.15210 (19)	0.2500	0.37930 (15)	0.0193 (4)	
C1	0.0383 (2)	0.2500	0.35335 (17)	0.0162 (4)	
H2	0.2584 (17)	0.485 (2)	0.3813 (13)	0.019*	
N2	0.28838 (12)	0.60009 (17)	0.38143 (10)	0.0155 (3)	
C2	0.0609 (2)	0.7500	0.38575 (17)	0.0191 (5)	
H2A	0.0277	0.8779	0.3825	0.029*	0.50
H2B	0.0299	0.6922	0.4503	0.029*	0.50
H2BA	0.0258	0.6800	0.3264	0.029*	0.50
C3	0.2103 (2)	0.7500	0.38283 (15)	0.0156 (4)	
C4	0.42343 (15)	0.6534 (2)	0.37939 (11)	0.0148 (3)	
C5	0.54317 (15)	0.5525 (2)	0.37848 (11)	0.0179 (3)	
H5	0.5432	0.4209	0.3774	0.022*	
C6	0.66199 (15)	0.6529 (2)	0.37921 (11)	0.0189 (3)	
H6	0.7460	0.5887	0.3797	0.023*	

### Atomic displacement parameters (Å^2^)

**Table:**

	*U*^11^	*U*^22^	*U*^33^	*U*^12^	*U*^13^	*U*^23^
S1	0.0159 (3)	0.0182 (3)	0.0287 (3)	0.000	−0.0020 (2)	0.000
N1	0.0179 (9)	0.0140 (9)	0.0260 (10)	0.000	0.0024 (7)	0.000
C1	0.0200 (10)	0.0105 (9)	0.0182 (10)	0.000	0.0034 (8)	0.000
N2	0.0169 (6)	0.0104 (6)	0.0191 (6)	−0.0018 (5)	0.0000 (5)	0.0004 (5)
C2	0.0170 (10)	0.0192 (11)	0.0211 (11)	0.000	0.0015 (8)	0.000
C3	0.0193 (10)	0.0151 (10)	0.0126 (9)	0.000	−0.0012 (8)	0.000
C4	0.0168 (7)	0.0141 (7)	0.0135 (6)	−0.0007 (6)	−0.0004 (5)	0.0004 (5)
C5	0.0215 (7)	0.0126 (7)	0.0197 (7)	0.0023 (6)	−0.0015 (6)	−0.0003 (6)
C6	0.0175 (7)	0.0200 (8)	0.0193 (7)	0.0026 (6)	−0.0007 (6)	0.0001 (6)

### Geometric parameters (Å, °)

**Table:**

S1—C1	1.628 (2)	C2—H2BA	0.9800
N1—C1	1.173 (3)	C4—C5	1.389 (2)
N2—C3	1.3289 (18)	C4—C4^i^	1.394 (3)
N2—C4	1.3888 (19)	C5—C6	1.379 (2)
N2—H2	0.881 (15)	C5—H5	0.9500
C2—C3	1.477 (3)	C6—C6^i^	1.401 (3)
C2—H2A	0.9800	C6—H6	0.9500
C2—H2B	0.9800		
			
N1—C1—S1	180.0 (2)	N2—C3—C2	125.52 (9)
C3—N2—C4	109.44 (13)	N2—C4—C5	132.32 (14)
C3—N2—H2	124.8 (12)	N2—C4—C4^i^	106.08 (8)
C4—N2—H2	125.7 (12)	C5—C4—C4^i^	121.60 (9)
C3—C2—H2A	109.5	C6—C5—C4	116.72 (15)
C3—C2—H2B	109.5	C6—C5—H5	121.6
H2A—C2—H2B	109.5	C4—C5—H5	121.6
C3—C2—H2BA	109.5	C5—C6—C6^i^	121.67 (9)
H2A—C2—H2BA	109.5	C5—C6—H6	119.2
H2B—C2—H2BA	109.5	C6^i^—C6—H6	119.2
N2—C3—N2^i^	108.97 (18)		

Symmetry codes: (i) *x*, −*y*+3/2, *z*.

### Hydrogen-bond geometry (Å, °)

**Table:**

*D*—H···*A*	*D*—H	H···*A*	*D*···*A*	*D*—H···*A*
N2—H2···N1	0.88 (2)	2.00 (2)	2.8627 (16)	168 (2)
